# Enhancing the Stability and Mobility of TFTs via Indium–Tungsten Oxide and Zinc Oxide Engineered Heterojunction Channels Annealed in Oxygen Ambient

**DOI:** 10.3390/nano14151252

**Published:** 2024-07-26

**Authors:** Seong-Hwan Lim, Dong-Gyun Mah, Won-Ju Cho

**Affiliations:** Department of Electronic Materials Engineering, Kwangwoon University, Gwangun-ro 20, Nowon-gu, Seoul 01897, Republic of Korea; 0325zxc@kw.ac.kr (S.-H.L.); madong13@kw.ac.kr (D.-G.M.)

**Keywords:** thin-film transistors, heterojunction, channel engineering, indium–tungsten oxide, zinc oxide, electron mobility, stability, charge trapping

## Abstract

This study demonstrates a significant enhancement in the performance of thin-film transistors (TFTs) in terms of stability and mobility by combining indium–tungsten oxide (IWO) and zinc oxide (ZnO). IWO/ZnO heterojunction structures were fabricated with different channel thickness ratios and annealing environments. The IWO (5 nm)/ZnO (45 nm) TFT, annealed in O_2_ ambient, exhibited a high mobility of 26.28 cm^2^/V·s and a maximum drain current of 1.54 μA at a drain voltage of 10 V, outperforming the single-channel ZnO TFT, with values of 3.8 cm^2^/V·s and 28.08 nA. This mobility enhancement is attributed to the formation of potential wells at the IWO/ZnO junction, resulting in charge accumulation and improved percolation conduction. The engineered heterojunction channel demonstrated superior stability under positive and negative gate bias stresses compared to the single ZnO channel. The analysis of O 1s spectra showed O_I_, O_II_, and O_III_ peaks, confirming the theoretical mechanism. A bias temperature stress test revealed superior charge-trapping time characteristics at temperatures of 25, 55, and 85 °C compared with the single ZnO channel. The proposed IWO/ZnO heterojunction channel overcomes the limitations of the single ZnO channel and presents an attractive approach for developing TFT-based devices having excellent stability and enhanced mobility.

## 1. Introduction

Recently, amorphous oxide semiconductor (AOS) thin-film transistors (TFTs) have attracted significant attention in various industries, including display, semiconductors, and sensors, owing to their excellent characteristics such as low processing temperature, large-area uniformity, and low leakage current [1,2,3,4,5,6]. Zinc oxide (ZnO) AOS is widely used in next-generation display technologies owing to its high optical transparency and the feasibility of forming smooth thin films on large-area substrates [7,8,9]. To enhance the properties of conventional ZnO, single AOSs incorporated with additional elements, such as indium, tungsten, and gallium, have been developed, resulting in materials such as zinc–indium oxide (ZIO), tungsten–indium–zinc oxide (WIZO), and indium–gallium–zinc oxide (IGZO) [10,11,12,13]. However, these materials have limited mobility, making it challenging to meet the requirements of specific applications [14,15]. High-mobility AOSs, such as indium–zinc oxide (IZO) and zinc oxynitride (ZnON), frequently suffer from insufficient bias stability, leading to a trade-off between mobility and stability in zinc-based AOSs [16,17,18].

To address these challenges, various approaches, such as optimizing annealing conditions and doping concentrations, forming metal capping layers, and developing heterojunction channel structures, are actively being studied [19,20,21,22]. Among these, heterojunction channel engineering has garnered significant attention for its potential to overcome the limitations of conventional AOS development [23,24,25]. In this study, we focused on enhancing the characteristics of ZnO channels through heterojunction engineering. Extensive research has been conducted on various factors affecting heterojunction AOS TFTs, including material types, channel thickness and ratios, and annealing conditions [26,27,28].

In this study, we explored the heterojunctions of indium–tungsten oxide (IWO) and ZnO, given the high indium content and excellent conductivity of IWO. Channels with various thickness ratios were fabricated based on a total AOS channel thickness of 50 nm. The fabricated heterojunction channels were subjected to an annealing process under different ambient conditions (O_2_ and air). We analyzed the electrical characteristics and mechanisms of heterojunction-channel TFTs based on the thickness ratios and ambient conditions. The mobility of IWO/ZnO heterojunction TFTs was found to increase, attributed to the potential well formed at the heterointerface. To validate this result, we conducted a series of systematic experiments. First, the optical bandgaps (*E*_g_) of ZnO and IWO were derived using UV–visible spectroscopy and Tauc plots, followed by the extraction of *E*_V_ − *E*_F_ (*E*_FV_) through X-ray photoelectron spectroscopy (XPS) valence band (VB) spectra [29,30]. Based on this, we investigated the alignment of the Fermi energy levels to confirm the formation of the potential well [31]. Moreover, various parameters were calculated and specifically compared based on the transfer curves. The IWO (5 nm)/ZnO (45 nm) channel annealed in O_2_ improved the on/off current ratio by more than 30 times to 7.50 × 10^9^ and *μ*_FE_ by more than 6 times to 26.28 cm^2^/V·s compared with the ZnO channel. Positive/negative gate bias stress tests were conducted on both the fabricated single and heterojunction channels to confirm the excellent stability of the IWO/ZnO heterojunction channel. An XPS O 1s analysis helped investigate the chemical bonding states of the IWO film depending on the annealing ambient conditions. This theoretical explanation elucidates the reasons behind the performance and stability improvements in the proposed heterojunction TFT. To ensure comprehensive reliability, additional stability evaluations were performed on both the optimal heterojunction channel and the single ZnO channel under various temperature conditions [32,33,34]. By analyzing the threshold voltage shifts in TFTs under bias and temperature stress, we confirmed the excellent charge-trapping characteristics and structural stability of IWO/ZnO. The IWO (5 nm)/ZnO (45 nm) heterojunction-channel TFT annealed in O_2_ ambient demonstrated remarkable improvements in terms of both mobility and stability compared with the single-ZnO-channel TFT. This advancement signifies notable progress in the development of ZnO channels through heterojunction engineering using IWO materials, contributing to the broader development of oxide semiconductor technology.

## 2. Materials and Methods

### 2.1. Material Specifications

We employed the following materials in the fabrication of the TFTs: a p-type Si substrate (orientation (100); resistivity range: 1–10 Ω·cm; LG Siltron Inc., Gumi, Republic of Korea), an IWO sputter target (In_2_O_3_:WO_3_ = 99:1 wt%, THIFINE Co., Ltd., Incheon, Republic of Korea), ZnO sputter targets (purity > 99.99%, THIFINE Co., Ltd.), and Al pellets (purity > 99.99%, THIFINE Co., Ltd.).

### 2.2. AOS Structure and Transistor Fabrication Process

**Figure 1:**
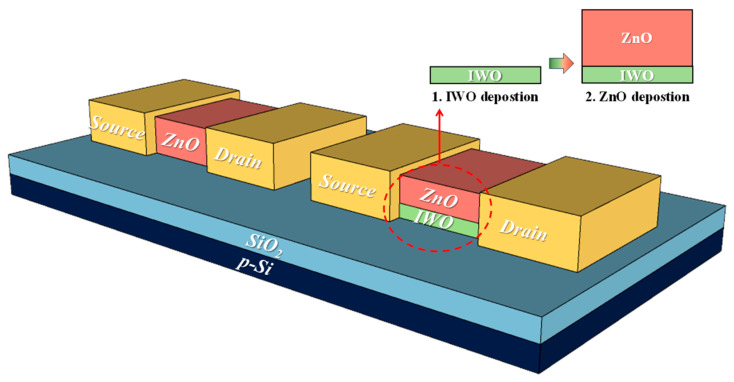
Schematic illustration of a TFT featuring a single ZnO channel and an IWO/ZnO heterojunction channel.

Figure 1 shows the schematics of the fabricated ZnO and IWO/ZnO TFTs. The substrate used was a heavily doped p-type Si wafer with a 100 nm thick thermally grown SiO_2_ layer. This was followed by the deposition of the AOS channel and source/drain on top of the substrate. ZnO and IWO were deposited using radio frequency (RF) magnetron sputtering. For the single-channel TFT, ZnO was deposited to a thickness of 50 nm. In the case of the heterojunction-channel TFTs, IWO with thicknesses of 5, 10, and 20 nm was used as the front channel, while ZnO with thicknesses of 45, 40, and 30 nm was deposited on the back, resulting in a total thickness of 50 nm. For heterojunction channels, IWO and ZnO were deposited through a continuous sputtering process to minimize the influence of impurities at the different AOS junction interfaces. The channel width and length were 120 µm and 60 µm, respectively. All the devices underwent a conventional thermal annealing process at 300 °C for 30 min under varying ambient conditions of air and oxygen. Al electrodes, 150 nm thick, were deposited using electron beam evaporation for the source/drain (S/D), and patterning was performed using the lift-off method. The width and length of the S/D electrodes were 150 µm and 120 µm, respectively.

### 2.3. Characterization Method

The transfer and output characteristics of the single- and heterojunction-channel TFTs were evaluated using an Agilent 4156B precision semiconductor parameter analyzer (Hewlett-Packard Co., Palo Alto, CA, USA). All the electrical measurements were conducted inside an electromagnetically shielded dark box to minimize external interference, such as noise signals and optical noise, at room temperature with a relative humidity of approximately 25%. Thermal instability evaluations were performed using a Cascade Microtech M150 probe station with an ERS AC3 chuck for temperature control. The thicknesses of the IWO, ZnO, and Al films were measured using a Dektak XT Bruker stylus profiler (Bruker, Hamburg, Germany). The optical transmittances of the films were measured using an Agilent 8453 UV–visible spectrophotometer (Hewlett-Packard Co., USA) over a wavelength range of 300–800 nm. Moreover, the film composition and chemical bonding states were investigated using a K-Alpha + XPS system (Thermo Fisher Scientific, Sunnyvale, CA, USA) with a monochromated Al X-ray source (Al Kα line: 1486.6 eV).

## 3. Results and Discussion 

### 3.1. Improvement in the Electron Mobility of Engineered Heterojunction AOS

To enhance the electron mobility in a single ZnO channel, heterojunction engineering was employed using an IWO front channel (known for its superior conductivity owing to a high indium content) and a ZnO back channel with comparatively lower conductivity. The junction between IWO and ZnO creates energy band bending that forms a potential well, facilitating electron accumulation and supporting enhanced mobility [35]. The thickness ratio of each AOS material in the heterojunction and the annealing ambient conditions play crucial roles in shaping the parameters observed in the TFT transfer curve [36]. The band diagram resulting from the IWO/ZnO junction was calculated to elucidate the mechanism behind mobility improvement through the heterojunction. The TFTs were fabricated with a single-ZnO-channel thickness of 50 nm, varying thickness ratios, and annealing ambient conditions for the IWO/ZnO heterojunction channel. A parameter analysis was conducted based on the transfer curve measurements, focusing on the conductivity characteristics extracted from threshold voltage values ranging from −1 to 1 V in the output characteristics.

**Figure 2:**
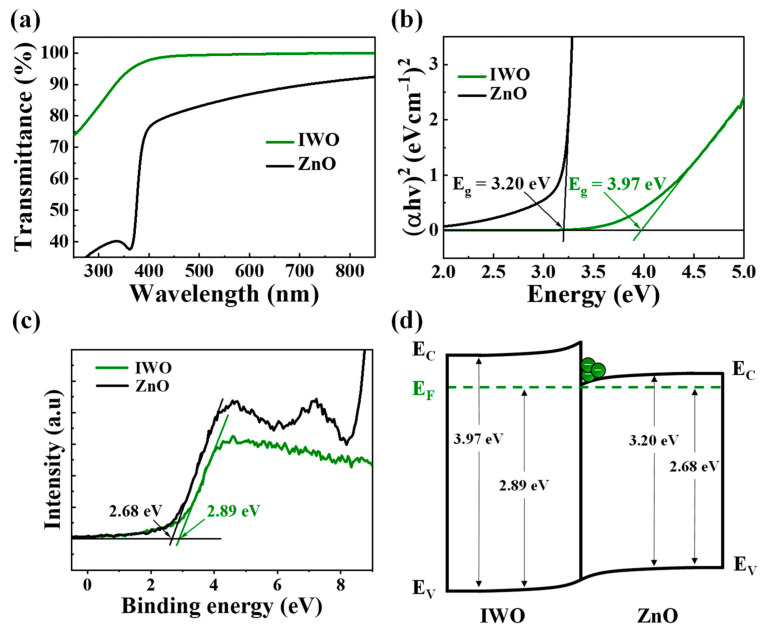
(**a**) Optical transmittance spectra; (**b**) plots of (*αhν*)^2^ versus *hν*; (**c**) XPS spectra showing the valence band edge; (**d**) energy band diagram for IWO and ZnO.

Figure 2a,b show the optical transmittance and (*αhν*)^2^ versus the hν plots for IWO and ZnO. *E*_g_ was determined using the Tauc plot method with Equations (1) and (2) [37,38].

(1) $$\alpha =\frac{1}{t}ln\left(\frac{1}{T}\right)$$

(2) $$\alpha h\nu ={\left(h\nu -E_{g}\right)}^{1/2}$$

where *α* is the absorption coefficient, *T* is the transmittance, and *t* is the film thickness. In the photon energy (*hν*), *h* is Planck’s constant, and *ν* is the frequency of the incident photon. The *E*_g_ values extracted for IWO and ZnO were 3.20 eV and 3.36 eV, respectively, extrapolated from the linear region of (*αhν*)^2^ versus *hν* where α = 0. Further, XPS VB spectra measurements (Figure 2c) provided *E*_FV_ values of 2.68 eV for IWO and 2.89 eV for ZnO, obtained from the intersection of the linearly fitted leading edge with the X-axis (binding energy). The energy band diagram of the heterojunction AOS (Figure 2d) shows that when IWO and ZnO form a junction, electrons migrate from IWO to ZnO, aligning with the overall Fermi energy levels. This results in the upward bending of the energy bands of IWO and the downward bending of ZnO, creating a potential well at the IWO/ZnO interface [39,40,41]. The accumulation of electrons in this potential well enhances the field-effect mobility by mitigating conductivity-limiting effects caused by traps at the insulator and channel interface, thereby significantly improving the percolation conduction properties [42,43].

**Figure 3:**
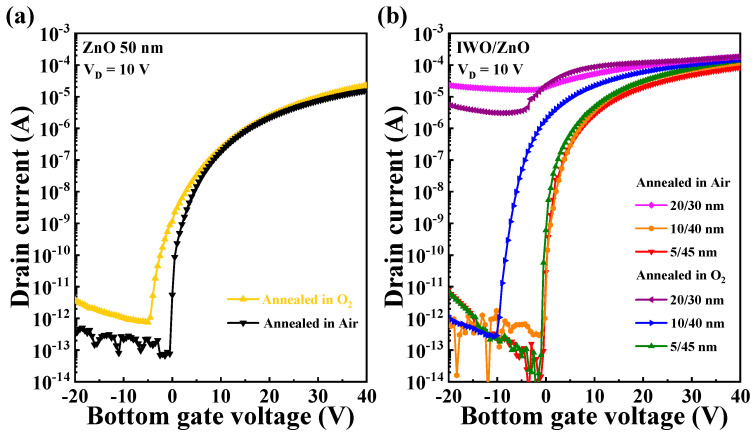
Transfer curves for (**a**) ZnO TFT and (**b**) IWO/ZnO TFT under different annealing ambient conditions.

Figure 3a,b show the transfer curves of single-channel and IWO/ZnO heterojunction-channel TFTs, each with a total thickness of 50 nm, under varying annealing ambient conditions (air, O_2_). The gate voltage (*V*_G_) was swept from −20 to 40 V, while the drain voltage (*V*_D_) was fixed at 10 V. Table 1 presents the key electrical performance parameters including the threshold voltage (*V*_th_), field-effect mobility (*µ*_FE_), subthreshold swing (*SS*), and on/off current ratio (*I*_ON/OFF_). Each extracted parameter represents the mean value, with the standard deviation falling within an acceptable range.

*V*_th_ was determined using the constant-current method normalized by the channel width and length, while *I*_ON/OFF_ was defined as the maximum on current divided by the minimum off current within the *V*_G_ sweep range. The field-effect mobility (*µ*_FE_) was calculated as follows [44]:

(3) $$\mu_{FE}=\frac{2L}{WC_{i}}{\left(\frac{\partial \sqrt{I_{D}}}{\partial V_{G}}\right)}^{2}$$

where *C*_i_ is the capacitance per unit area, *W* is the channel width, and *L* is the channel length. The subthreshold swing (*SS*) was determined as follows [45]:

(4) $$S.S=\frac{{\partial V}_{G}}{\partial logI_{D}}$$

When comparing the *V*_th_ values, the ZnO TFT exhibited a *V*_th_ of 0.48 V in air ambient, demonstrating superior characteristics compared with that observed under O_2_ ambient (−2.65 V). Meanwhile, for the heterojunction channel in the front IWO thickness/back ZnO thickness (annealed ambient) configurations of 10/40 (O_2_), 10/40 (air), 5/45 (O_2_), and 5/45 (air), the *V*_th_ values were −8.37, 0.25, −0.44, and 0.19 V, respectively. In particular, the 20/30 channel configuration showed limited on/off operation due to the high conductivity of the 20 nm thick IWO layer, regardless of the post-annealing ambient in O_2_ or air. Except for the 20/30 (O_2_), 20/30 (air), and 10/40 (O_2_) configurations, all configurations exhibited *V*_th_ characteristics within the range of −1 to 1 V. In terms of *µ*_FE_, ZnO (O_2_), ZnO (air), 10/40 (O_2_), 10/40 (air), 5/45 (O_2_), and 5/45 (air) demonstrated values of 7.14, 3.8, 26.54, 28.89, 26.28, and 18.24 cm^2^/V·s, respectively. This highlights the significant enhancement in *µ*_FE_ for the IWO/ZnO heterojunction channel compared with the single ZnO channel. In particular, the 5/45 (O_2_) configuration exhibited an *I*_ON/OFF_ of 7.50 × 10^9^ and an *SS* of 0.17 V/dec, indicating a superior electrical performance across all the extracted parameters.

**Figure 4:**
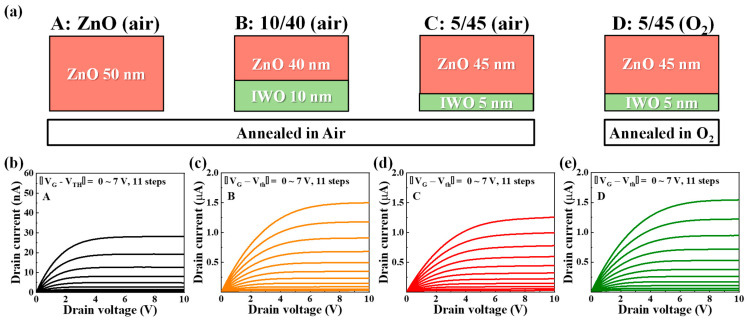
(**a**) Schematic representation of channel structures and annealing conditions for selected TFTs. Output curve characteristics for (**b**) ZnO (air) [A], (**c**) IWO/ZnO (10 nm/40 nm, air) [B], (**d**) IWO/ZnO (5 nm/45 nm, air) [C], and (**e**) IWO/ZnO (5 nm/45 nm, O_2_) [D].

Based on a comprehensive analysis of the parameters extracted from Figure 3, additional electrical characteristic analyses were conducted for ZnO (air), 10/40 (air), 5/45 (O_2_), and 5/45 (air) TFTs. Figure 4a shows the schematic of the channel structures for the selected TFTs. For convenient classification, ZnO (air) is denoted by A, 10/40 (air) is denoted by B, 5/45 (air) is denoted by C, and 5/45 (O_2_) is denoted by D. Figure 4b–e present the output curves of the selected channels (A–D). *V*_D_ was swept from 0 to 10 V, and |*V*_G_–*V*_th_| was varied in 11 steps from 0 to 7 V. All four types of TFTs exhibited clear linear and saturation regions in the output curves, with the maximum saturation currents being 28.08 nA (A), 1.50 μA (B), 1.26 μA (C), and 1.54 μA (D), respectively. The *I*_D_ value of the heterojunction channels was more than 30 times higher than that of the single channel. The high *I*_D_ in the heterojunction TFTs indicates better driving capability compared with the single-channel TFT, consistent with the improved mobility inferred from the transfer curves.

### 3.2. Stability of Heterojunction AOS under Gate Bias Stress

Evaluating the instability of TFT devices under gate bias stress is essential for understanding the potential degradation characteristics such as the threshold voltage shift (Δ*V*_th_), mobility reduction, and increased leakage current in the proposed heterojunction channels [46]. This is a critical factor for long-term operation and practical applications. Therefore, we conducted stability evaluations under positive and negative gate bias stresses for TFTs of types A, B, C, and D. Moreover, the physical mechanisms behind the Δ*V*_th_ characteristics of the transfer curves under the gate bias stress are clearly explained through the analysis of the O 1s spectra.

**Figure 5:**
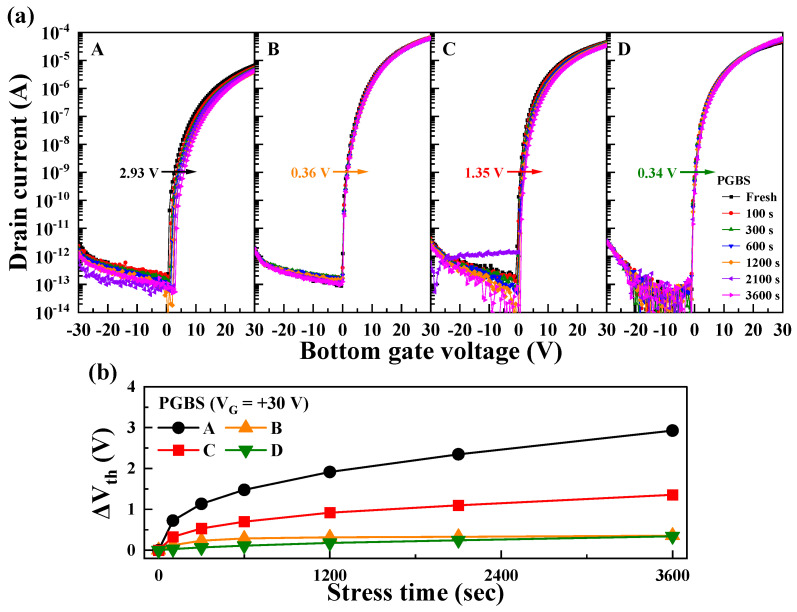
(**a**) Transfer curve instability of ZnO and IWO/ZnO TFTs under positive gate bias stress (PGBS) with *V*_G_ = + 30 V; (**b**) variation in the threshold voltage (*V*_th_) as a function of the stress time.

Figure 5a shows the changes in the transfer curves of TFTs with channel structures A–D under a positive gate bias stress (PGBS). The PGBS was applied at *V*_G_ = 30 V for 3600 s, and the transfer curves were measured by sweeping *V*_G_ from −30 to 30 V. Figure 5b shows Δ*V*_th_ over the stress time during PGBS application. With the increase in the stress time, the *V*_th_ values of all the TFTs shifted in the positive direction. Heterojunction-channel TFTs generally exhibited superior *V*_th_ stability compared with single-channel TFTs. The single ZnO channel (A) showed a Δ*V*_th_ of 2.93 V, while the heterojunction channels showed Δ*V*_th_ values of 0.36 V (B), 1.35 V (C), and 0.34 V (D), respectively. Under PGBS conditions, Δ*V*_th_ is primarily attributed to electron trapping at the interface between the channel and gate insulator [47,48]. The conductive IWO layer, rich in indium content, has a high electron concentration. Consequently, numerous electrons in the IWO already occupy interfacial trap sites during PGBS. This phenomenon resulted in high *V*_th_ stability for B. For C, the reduction in the IWO thickness from 10 to 5 nm resulted in greater Δ*V*_th_ instability. This is interpreted as a consequence of the relative decrease in the electron concentration, leading to an increase in the unoccupied interfacial trap sites. However, the D TFT showed significantly improved PGBS stability despite the IWO layer thickness being 5 nm. This is interpreted as a result of oxygen annealing improving the interface between the channel and dielectric layers, as well as reducing impurities such as OH^−^ within the IWO channel, thereby decreasing the interfacial trap sites that can be occupied by electrons. This is explained according to the O 1s spectra analysis obtained from XPS.

**Figure 6:**
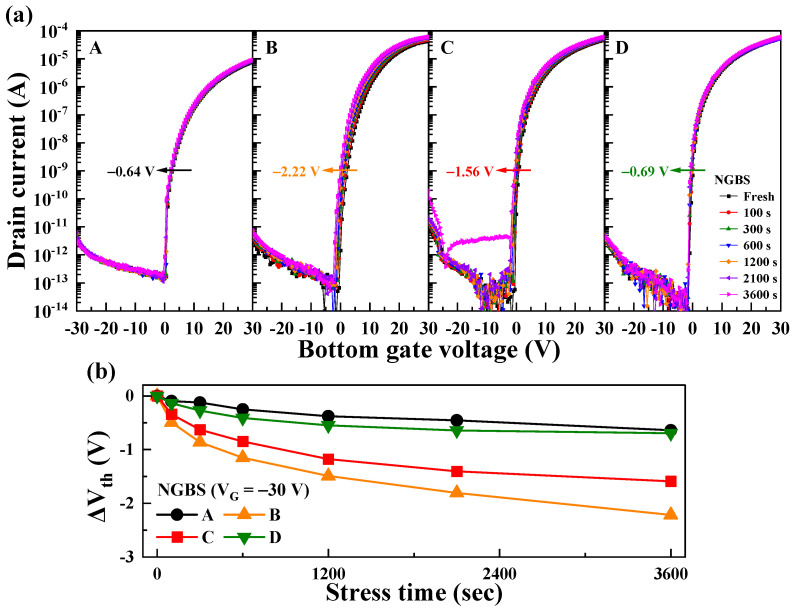
(**a**) Instability of the transfer curves for ZnO and IWO/ZnO TFTs under NGBS with *V*_G_ = −30 V; (**b**) variation in *V*_th_ as a function of the stress time.

**Figure 7:**
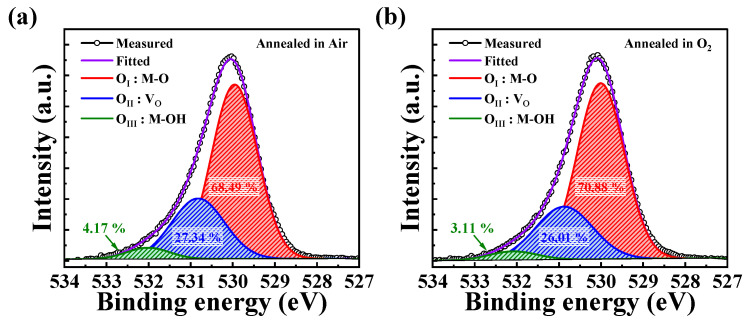
XPS analysis of the O 1s spectra of IWO films under different annealing ambient conditions: (**a**) air and (**b**) O_2_.

The impact of heterojunction channel types on the stability under negative gate bias stress (NGBS) was also investigated. Figure 6a illustrates the changes in the transfer curves of TFTs with channels A–D under NGBS conditions. NGBS was applied at *V*_G_ = −30 V for 3600 s, with *V*_G_ swept from −30 to 30 V. Figure 6b shows Δ*V*_th_ during the NGBS. Channel A showed a Δ*V*_th_ of −0.64 V, while heterojunction channels B, C, and D recorded Δ*V*_th_ values of −2.22, −1.56, and −0.69 V, respectively, demonstrating varying stability depending on the channel type. Δ*V*_th_ during NGBS can be primarily attributed to the additional free electrons caused by oxygen vacancy defects near the channel and gate insulator interface [49,50]. Under NGBS, as holes accumulate at the interface, oxygen vacancies (V_O_) transition to V_O_^1+^ or V_O_^2+^, releasing electrons (V_O_ + h^+^ ⟶ V_O_^1+^ + e^−^ or V_O_ + 2h^+^ ⟶ V_O_^2+^ + 2e^−^) [51]. This process increases the electron concentration in the channel layer, consequently shifting *V*_th_ in the negative direction. In B, the presence of V_O_ in the interfacial IWO layer was a major factor in reducing NGBS stability by generating more doubly ionized oxygen vacancies (V_O_^2 +^) in the channel under NGBS conditions. As the thickness of the front IWO layer decreased in C, the reduction in the front IWO layer thickness led to a decrease in V_O_, thereby improving the NGBS stability. D, annealed in oxygen, exhibited reduced V_O_ compared with C, which was annealed in air, demonstrating the highest NGBS stability among the fabricated heterojunction channels. The results regarding *V*_O_ under the annealing ambient conditions are explained in Figure 7.

As part of the mechanism analysis of the Δ*V*_th_ results under gate bias stress, we conducted an XPS analysis of IWO thin films under different annealing ambient conditions (air, O_2_). Figure 7a,b show the O 1s spectra obtained from the XPS analysis. These spectra can be divided into three subpeaks, designated as O_I_, O_II_, and O_III_, centered at binding energies of 530.1 ± 0.2 eV, 530.8 ± 0.2 eV, and 532.1 ± 0.2 eV, respectively, using Gaussian fitting [52,53,54]. Each peak corresponds to different chemical states, namely metal–oxygen (M–O) bonds, oxygen vacancies (V_O_), and impurities such as hydroxyl groups (-OH), which are known as trapping sites at the interface [55]. The IWO layer annealed in O_2_ showed an increase in the M–O bond ratio from 68.49% to 70.88% compared with the IWO layer annealed in air. Moreover, the ratios of V_O_ and -OH decreased from 27.34% to 26.01% and from 4.17% to 3.11%, respectively. As a result, an analysis of the O 1s spectra from the D TFT annealed in O_2_ ambient indicates that the increase in the M–O concentration enhances the percolation conduction path, thereby improving mobility [56,57]. The reduction in V_O_ decreases electron emission, enhancing the stability under NGBS conditions. Further, a decrease in the -OH concentration indicates a reduction in the number of impurities within the channel layer, contributing to excellent stability under PGBS conditions [58]. 

### 3.3. Reliability Evaluation of Heterojunction Channel under Bias Temperature Stress

**Figure 8:**
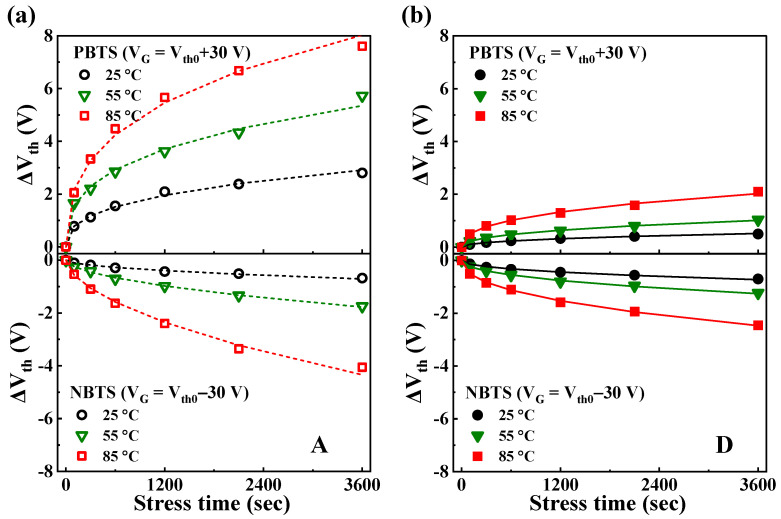
Temperature dependence of Δ*V*_th_ under PBTS (*V*_G_ = *V*_th0_ + 30 V) and NBTS (*V*_G_ = *V*_th0_ − 30 V) tests for (**a**) A and (**b**) D devices, conducted at 25, 55, and 85 °C. The curves were fitted using the stretched-exponential equation.

Based on comprehensive evaluation results, heterojunction channel D demonstrated excellent performance as a transistor through process optimization and exhibited outstanding stability even under high voltage stress. To further assess the reliability of the D TFT, we conducted a thermal stability test under bias. Since thermal stress significantly affects electron mobility and trap states, it ensures the long-term reliability of the TFT [59]. In this study, we detailed the effects of charge trapping and defects under high-voltage negative and positive bias temperature stresses. Figure 8a,b present the Δ*V*_th_ values of A and D over time under positive bias temperature stress (PBTS) and negative bias temperature stress (NBTS) at various temperatures. The PBTS and NBTS tests were conducted at 25, 55, and 85 °C with *V*_G_ = *V*_th0_ ± 30 V for 3600 s, where *V*_th0_ represents the initial *V*_th_ before applying the gate bias stress. The Δ*V*_th_ values of all the TFTs increased proportionally with time and temperature. During PBTS, the Δ*V*_th_ values for A and D were measured to be 2.80 and 0.50 V at 25 °C, 5.72 and 1.04 V at 55 °C, and 7.60 and 2.03 V at 85 °C, respectively. During NBTS, the Δ*V*_th_ values for A and D were −0.68 and −0.70 V at 25 °C, −1.75 and −1.26 V at 55 °C, and −4.05 and −2.47 V at 85 °C, respectively. As a result, A exhibited a high increase rate of Δ*V*_th_ with increasing stress temperature, whereas D maintained a relatively stable Δ*V*_th_ even under high-temperature stress conditions. Notably, under NBTS conditions, both channels exhibited similar Δ*V*_th_ at 25 °C, but as the temperature increased to 55 °C and 85 °C, the Δ*V*_th_ value of A increased significantly. This indicates that heterojunction channel D provides excellent stability under thermal stress. The experimental data for Δ*V*_th_ were fitted using a stretched-exponential equation, defined as follows [60,61]:

(5) $${\Delta V}_{th}\left(t\right)={\Delta V}_{th0}\left\{1-exp\left[-{\left(\frac{t}{\tau }\right)}^{\beta }\right]\right\}$$

where Δ*V*_th0_ is Δ*V*_th_(*t*) at infinite time; *β* is the stretched-exponential exponent; *t* is the stress time; and *τ* represents the characteristic charge-trapping time from the channel to the dielectric layer, which depends on the temperature. The dotted line in Figure 8a and the solid line in Figure 8b are fitted using the stretched-exponential equation, showing a good agreement with the experimental results. This suggests that Δ*V*_th_ in the gate bias temperature stress evaluation is due to a thermally activated charge-trapping mechanism. 

**Figure 9:**
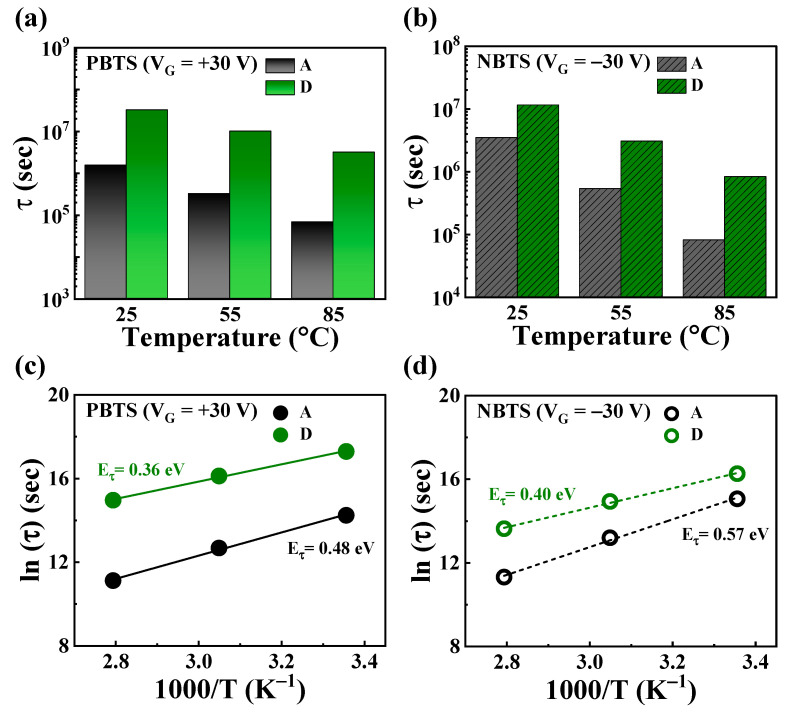
Box plots of the charge-trapping time (*τ*) for A and D during (**a**) PBTS and (**b**) NBTS. Arrhenius plots of ln(*τ*) versus the reciprocal of the temperature during (**c**) PBTS and (**d**) NBTS tests.

Figure 9a,b depict the *τ* values extracted from the time-dependent Δ*V*_th_ during the PBTS and NBTS tests shown in Figure 8a,b; *τ* represents the time for carriers to become trapped within the insulator or at the interface between the channel and insulator. In the stretched-exponential equation, *τ* for thermally activated carriers is expressed as follows:

(6) $$\tau =\tau_{0}exp\left(\frac{E_{\tau }}{k_{B}T}\right)=\nu^{-1}exp\left(\frac{E_{\tau }}{k_{B}T}\right)$$

where the thermal activation energy is denoted by *E*_a_ = *E_τ_β*, with *E_τ_* representing the average effective energy barrier that electrons in the channel must overcome before entering the gate insulator [62]. Here, *τ*_0_ is the thermal pre-factor, and *ν* is the frequency pre-factor for emission over the barrier.

Table 2 presents the values of *τ* and *β* calculated using this equation. During PBTS and NBTS, Device D exhibited significantly higher *τ* values across all the tested temperatures. Specifically, at 25 °C, the *τ* values were 21 and 3 times higher for D compared with A. At 55 °C, they were 31 and 5 times higher, and at 85 °C, they were 46 and 10 times higher. While *τ* values gradually decreased with increasing test temperature for both devices, the decrease was more pronounced in Device A. This suggests potential long-term reliability issues in Device A due to rapid carrier trapping.

Figure 9c,d present the Arrhenius plots of ln(*τ*) versus the reciprocal of the absolute temperature (1/*T*) for TFTs in Device A and Device D during PBTS and NBTS tests. The linear relationship between ln(*τ*) and 1/*T* indicates a thermally activated charge-trapping process. In these plots, the slope corresponds to *E_τ_* for charge transport. Under PBTS, A and D exhibited *E_τ_* values of 0.36 eV and 0.48 eV, respectively, while under NBTS, these values were 0.40 eV and 0.57 eV, respectively. Lower *E_τ_* values are associated with fewer defects and a more ordered channel structure, suggesting that heterojunction channel D can mitigate charge-trapping issues and enhance device reliability [63].

Finally, as part of the evaluation of the enhanced performance and reliability of the proposed optimal heterojunction TFT, a comparison was conducted with reported single-channel IWO and ZnO TFTs. Table 3 presents the key electrical performance parameters and gate bias stability characteristics of the single-channel IWO and ZnO TFTs deposited through sputtering, as well as the heterojunction TFTs fabricated in this study. To ensure reliable results in the gate bias stability evaluation, the Δ*V*_th_ values for each group were compared under similar stress durations (approximately 3600 s). Compared to all single-channel TFTs, the proposed heterojunction TFT showed competitive mobility (26.28 cm^2^/V·s) and excellent stability (Δ*V*_th_ under PGBS = 0.34 V, Δ*V*_th_ under NGBS = −0.97 V). In particular, compared to single-channel ZnO TFT, the mobility was more than double, and the Δ*V*_th_ values were significantly lower, indicating superior reliability.

## 4. Conclusions

Single-phase ZnO channels are associated with limitations in terms of their electron mobility and stability. To address these issues, we engineered heterojunction channels using IWO/ZnO structures, resulting in significant performance improvements. First, energy band diagrams were computed for IWO and ZnO junctions, elucidating the mechanism behind the improved electron mobility in the IWO/ZnO heterojunction channel. The extracted parameters from the transfer curves (*µ*_FE_ = 26.28 cm^2^/V·s, *I*_ON/OFF_ = 7.5 × 10^9^, *SS* = 0.17 V/dec) demonstrated substantial performance improvements compared with single ZnO channels, corroborating theoretical expectations. Further, under PGBS testing, the maximum Δ*V*_th_ values for IWO/ZnO and single ZnO were 0.34 V and 2.93 V, respectively, highlighting a significant difference of 2.59 V, which is crucial for the reliability of TFTs. Moreover, an O 1s XPS analysis of IWO films under varied annealing ambient conditions distinctly illustrated the basis for superior mobility and stability in the optimized heterojunction channel. Finally, an analysis of the parameters derived from bias temperature stress evaluations confirmed the superior charge-trapping characteristics and structural stability within the channel compared with the single ZnO channels.

In conclusion, the IWO/ZnO heterojunction channel exhibited promising potential for advancing high-performance oxide semiconductors, overcoming the mobility and stability limitations encountered in both single ZnO channels and previously known oxide semiconductors.

## Figures and Tables

**Table 1:** Variations in the threshold voltage, mobility, and on/off current ratio under different annealing ambient conditions and channel thicknesses.

Channel Type (Ambient)	*V*_th_ [V]	*μ*_FE_ [cm^2^/V·s]	*S.S* [V/dec]	*I*_ON/OFF_ [A/A]
ZnO (O_2_)	−2.65	7.14	0.72	1.61 × 10^7^
[A] ZnO (Air)	0.48	3.80	0.27	2.04 × 10^8^
10/40 (O_2_)	−8.37	26.54	0.54	4.06 × 10^8^
[B] 10/40 (Air)	0.25	28.89	0.27	5.03 × 10^8^
[C] 5/45 (Air)	0.19	18.24	0.20	5.61 × 10^9^
[D] 5/45 (O_2_)	−0.44	26.28	0.17	7.50 × 10^9^

**Table 2:** Charge-trapping time (*τ*) and stretched-exponential exponent (*β*) extracted from Δ*V*_th_ measurements for TFTs A and D.

Temperature [°C]	PBTS				NBTS			
	A		D		A		D	
	*τ*	*β*	*τ*	*β*	*τ*	*β*	*τ*	*β*
25	1.5 × 10^6^	0.38	3.2 × 10^7^	0.54	3.5 × 10^6^	0.44	1.2 × 10^7^	0.46
55	3.2 × 10^5^	0.36	1.0 × 10^7^	0.56	5.4 × 10^5^	0.43	3.0 × 10^6^	0.47
85	6.7 × 10^4^	0.39	3.1 × 10^6^	0.58	8.2 × 10^4^	0.41	8.4 × 10^5^	0.45

**Table 3:** Comparison of key electrical performance parameters and gate bias stability of heterojunction and single-channel TFTs from the literature and this work.

Channel Layer	Gate Insulator /Thickness [nm]	*V*_th_ [V]	*μ*_FE_ [cm^2^/V·s]	*S.S* [V/dec]	Δ*V*_th_ under PGBS [V]	Δ*V*_th_ under NGBS [V]	Ref.
ZnO	SiO_2_/100	4.3 ± 0.5	11.5 ± 1.3	0.65 ± 0.08	8.7 ± 0.8	−9.6 ± 0.9	[64]
ZnO	SiO_2_/150	15	8.1	1.35	3	N/A	[65]
ZnO	SiO_2_/100	4.1 ± 0.6	10.5 ± 0.5	0.45 ± 0.07	2.7	−3.2	[66]
IWO	SiO_2_/100	−3.4	36.7	0.39	≈2.5	N/A	[67]
IWO	SiO_2_/100	0.5	27.55	0.5	4.5	−0.92	[68]
IWO	SiO_2_/100	−0.5	21.7	0.47	N/A	N/A	[69]
[D] IWO/ZnO	SiO_2_/100	−0.44	26.28	0.17	0.34	−0.97	This work

## Data Availability

Data are contained within the article.
